# The “Sifting of Our Materia Medica:” a Clinical Illustration

**Published:** 1884-11

**Authors:** Ad. Lippe

**Affiliations:** Philadelphia


### THE "SIFTING OF OUR MATERIA MEDICA:" A CLINICAL ILLUSTRATION.

AD. LIPPE, M. D., PHILADELPHIA.

The case here referred to has been laid before the profession many years ago, but at this time, when a great effort is going to be made to destroy the usefulness of our materia medica by means of an indiscriminate sifting process, a little illustration may serve to cause some thoughtful colleagues to reflect before they progress with and go into the destruction-bringing sifting-trap.

The patient was a boy about fifteen years old, who was in the twenty-first day of typhus abdominalis. Dr. H. N. Guernsey had been in attendance and had treated him in his always careful manner, when the following prominently characteristic symptoms were observed: The whole alimentary canal and organs were in a state of ulceration; his mouth especially was extremely painful; could not even allow fluids to touch his mouth without complaining severely; he vomited blood-streaked mucus, and had severe diarrhœa; sleepless; during the night he would shriek out violently without being able to state the cause of it. Nitr. ac., Mercury, Arum triphyllum had had no effect on him. The mental symptoms called our attention to Stramonium; but here was that last tormenting symptom, the sore mouth, and as we were in doubt whether Stramonium could be administered under the strict law of the similars, we resolved to study up the case before prescribing. In the admirable rendition of Stramonium by the ever painstaking C. Hering we found symptom 1023: "It feels as if the inner mouth were raw and sore (Hahnemann)"—and in going as a last resort, as is our habit, to the *Materia Medica Pura* of Hahnemann, we there found that symptom (20) an observation of Hahnemann himself. After further comparisons, we were satisfied that Stramonium was the similar remedy, and a few doses of it (high potencies) were administered with the hoped-for result—a speedy cure. The prover had only a sensation (feeling) as if the mouth were raw and sore; the patient had that sensation also, and additionally had really a raw and sore mouth. The result proved that our interpretation of that single apparently unimportant symptom, observed by one prover only, was correct.

A revision of the materia medica is now seriously proposed, and the American Institute has taken the initial steps for the "revise."

The Bureau of Materia Medica, through its Chairman, Dr. J. P. Dake, presented, at the last meeting of the American Institute, a report, and read a schema for the revision and publication of a purified materia medica, sifting out that which is worthless and arranging the whole in a condensed form.

The schema is objectionable on various grounds; for instance, rule 5 reads, "*Include as a rule no drug that has not shown pathogenetic* (*would read better* SICKMAKING) *power in two or more provers.*" What would have become of the case above referred to if violent hands were laid on "singly observed symptoms"?

No. 10 was properly objected to by thinking members. It reads: 10. "*Include no symptoms reported as coming from attenuations above the twelfth decimal only when in accord with symptoms from attenuations below.*" No. 10 is objectionable. It is illogical, to begin with, if it is admitted that attenuations above the twelfth can cause symptoms. Why then reject those not in accord with those observed from attenuations up to the twelfth? Does the learned gentleman, who committed himself unwittingly to the acknowledgment that attenuations above the twelfth can have any sickmaking powers, not know that the higher potencies are more potent in every respect than the twelfth? Does it not follow that on that very account these higher attenuations (potencies?) will cause, in many instances, more characteristic symptoms than the twelfth develops? The arbitrary limitation to the twelfth attenuation is unwarranted; or does it seem needful in these days to disregard the teachings and statements of the father of our school? What will the revisers do with Theridion curassavum, which has only been proved in the thirtieth decimal potency? Will they throw it overboard and insult every healer who has reported cases cured by Theridion? Furthermore, there are now a not inconsiderable number of characteristic symptoms of acknowledged value observed only by provings with high potencies. Will the makers of the revised and purified materia medica throw them out? *They will if they know them!* But alas! they do not know much about the way the materia medica was obtained; we do, and if that promised *Opus* should see the light of day it may give us great pleasure to expose these purifiers, especially if they have admitted symptoms only obtained from and only cured by high potencies.

11. *Omit the contributions of Hahnemann and his fellow-provers to the Materia Medica Pura and Chronic Diseases, which are already accessible to the profession, and of which we do not*

*possess the day books.* The play of *Hamlet* with Hamlet left out! Why reject that immortal work of Hahnemann? *Because we do not possess the day books!* For the same reason, Hering's provings may be set aside as well as other provings. It is an old complaint made by Dr. Richard Hughes and his followers that we have not Hahnemann's day books. Will these everlasting fault-finders take up Hahnemann's *Materia Medica Pura* and his *Chronic Diseases*, and for their own satisfaction write out the day books! If they desire to have the day books they are there, can be obtained easily if they observe on what day the various provers observed the symptoms. If they write them out as far as given, the day books are ready. Hahnemann gave the name of the provers, and the abbreviations of their names are given. To many a symptom is added the day on which it was observed. When Hahnemann performed the herculean work of *creating* a pure materia medica it was very difficult to find a publisher, and his ardent friend who was fully convinced of the great truth of Homœopathy, the bookseller Arnold, at Dresden, published these works at a very great pecuniary sacrifice, and now, some sixty years later, it is claimed that he should have published "day books" also. Fault-finders who have not the remotest idea of the magnitude of Hahnemann's and his publisher's sacrifices. In our days we find published "new remedies" without "sacrifices," and these fault-finders never mention these innovations adversely. What was the corner-stone of our school? Why, Hahnemann's works.

Once more we return to the case above related. From the facts there stated different men who have differing views of Homœopathy will make different uses, draw different deductions. The thoughtful healer will mark in his *Materia Medica* opposite the symptom related "Confirmed" (sore mouth and alimentary canal in typhus healed). The man who rides the pathological hobby-horse will draw the deduction that Stramonium will heal sore mouth and ulcerated alimentary canals in every case of typhus fever, and he will be sadly disappointed if he leaves out "*the mental symptoms*," as related in this case. Natrum sulfuricum has healed similar pathological conditions, so have Nitr. ac., Arum tri., Argt. nitr. The true healer considers the "totality of symptoms," not a hypothetical pathological condition. He individualizes and does not generalize, guided by a pathological picture-book; he also does not reject a symptom because it was only observed by one prover; nor does he ever dream of labor-saving books, such as the condensing, purifying divisions, with the twelfth dilution as a standard, propose to publish. If the *Opus* really comes out there will be found among the "veterans" a goodly number who will write "A Criticism." These old veterans did utilize Hahnemann's *Materia Medica Pura* and his *Chronic Diseases*, and there were many among them who learned the German language that they might have access to this indispensable work, as many years elapsed before a translation into the English and French languages appeared, while still later it was translated into almost all languages. With Hahnemann's great unparalleled *Materia Medica Pura* in hand, the battle against the common school of medicine, with their materia medica bristling with materialism and arbitrary classifications, began, and by the aid of Hahnemann's works battles were won; Homœopathy was established because it cured. Would not men endowed with a reasonable amount of common sense leave the foundation on which was built our school intact and be content with adding to the inheritance left us. I well remember to have seen a copy of Hahnemann's *Materia Medica Pura* and his *Chronic Diseases* at the residence of the late Dr. Bousquet, at Havana, in 1856. Thumbed as these volumes were, their soiled condition gave evidence of their frequent use, and this old Frenchman had introduced Homœopathty into Cuba; the yellow fever and the cholera were by him cured, by means of the *Materia Medica* unabridged and unrevised, and with the thirtieth potency. Later this good healer learned to use higher potencies, and Homœopathy was by him permanently established and used by an intelligent community. Many similar cases have come to my knowedge. The early practitioners in all parts of the world established and made respected our school by just these means. The English reading homœopaths will continue to use the superior translation of our *Hahnemannian Materia Medica* by Dr. Drysdale, far superior to the slovenly translation by the late J. C. Julius Hempel, who had even omitted that greatest paper of the Master, "The Genius of the Homœopathic Healing Art," which paper we take the liberty to recommend for study to men now deliberating how to "condense and purify" our own materia medica before they present to the world "*a Caricature*."

---

BRITISH JOURNAL OF HOMŒOPATHY.—This able and old homœopathic medical journal will cease its welcome visits after this year. It is a great misfortune for Homœopathy that its journals are not better supported by subscriptions and contributions to its pages.

